# Is volunteering a public health intervention? A systematic review and meta-analysis of the health and survival of volunteers

**DOI:** 10.1186/1471-2458-13-773

**Published:** 2013-08-23

**Authors:** Caroline E Jenkinson, Andy P Dickens, Kerry Jones, Jo Thompson-Coon, Rod S Taylor, Morwenna Rogers, Clare L Bambra, Iain Lang, Suzanne H Richards

**Affiliations:** 1 Primary Care, University of Exeter Medical School, Smeall Building, St Luke’s Campus, Exeter EX1 2LU, UK; 2 Public Health, Epidemiology and Biostatistics, School of Health and Population Sciences, University of Birmingham, Edgbaston, Birmingham B15 2TT, UK; 3 PenCLAHRC, University of Exeter Medical School, Veysey Building, Salmon Pool Lane, Exeter EX2 4SF, UK; 4 Department of Geography, Wolfson Research Institute for Health and Wellbeing, Durham University, Queen’s Campus, Stockton-on-Tees TS17 6BH, UK

**Keywords:** Volunteering, Community participation, Systematic review, Meta-analysis, Health, Outcomes, Mortality

## Abstract

**Background:**

Volunteering has been advocated by the United Nations, and American and European governments as a way to engage people in their local communities and improve social capital, with the potential for public health benefits such as improving wellbeing and decreasing health inequalities. Furthermore, the US Corporation for National and Community Service Strategic Plan for 2011–2015 focused on increasing the impact of national service on community needs, supporting volunteers’ wellbeing, and prioritising recruitment and engagement of underrepresented populations. The aims of this review were to examine the effect of formal volunteering on volunteers’ physical and mental health and survival, and to explore the influence of volunteering type and intensity on health outcomes.

**Methods:**

Experimental and cohort studies comparing the physical and mental health outcomes and mortality of a volunteering group to a non-volunteering group were identified from twelve electronic databases (Cochrane Library, Medline, Embase, PsychINFO, CINAHL, ERIC, HMIC, SSCI, ASSIA, Social Care Online, Social Policy and Practice) and citation tracking in January 2013. No language, country or date restrictions were applied. Data synthesis was based on vote counting and random effects meta-analysis of mortality risk ratios.

**Results:**

Forty papers were selected: five randomised controlled trials (RCTs, seven papers); four non-RCTs; and 17 cohort studies (29 papers). Cohort studies showed volunteering had favourable effects on depression, life satisfaction, wellbeing but not on physical health. These findings were not confirmed by experimental studies. Meta-analysis of five cohort studies found volunteers to be at lower risk of mortality (risk ratio: 0.78; 95% CI: 0.66, 0.90). There was insufficient evidence to demonstrate a consistent influence of volunteering type or intensity on outcomes.

**Conclusion:**

Observational evidence suggested that volunteering may benefit mental health and survival although the causal mechanisms remain unclear. Consequently, there was limited robustly designed research to guide the development of volunteering as a public health promotion intervention. Future studies should explicitly map intervention design to clear health outcomes as well as use pragmatic RCT methodology to test effects.

## Background

The UN [1] defines volunteering as an act of free will that results in benefits to others (e.g. individuals, groups, the environment) outside of, or in addition to support given to close family members. While these criteria are widely accepted [2], there is considerable debate as to whether volunteering is associated with benefits, either paid or otherwise, to the volunteer. The UN definition does allow some financial reimbursement of direct expenses accrued while volunteering.

Worldwide, the prevalence of adult volunteering varies considerably with estimates of 27% in the USA [3], 36% in Australia [4], and 22.5% in Europe (country range: 10% to >40%) [5]. Although volunteering is widespread, substantial social and health inequalities exist; people from more deprived social backgrounds [6,7] or people reporting long-term chronic health conditions are much less likely to volunteer than their wealthier and healthier counterparts [8]. The main reason given for volunteering tends to be altruistic, such as to ‘give something back’ to their community, or to an organisation or charity that has supported them in some way [9]. Other reasons include improving employment opportunities, widening social circles or using the activity as a distraction from problems in their daily life [9]. Health improvement is rarely cited as a motive to volunteer, yet there is a popular policy perception that volunteering is associated with improved health and wellbeing [10-14].

In 2010, the UK government launched the ‘Building the Big Society’ policy [15] which called for low cost, sustainable interventions, such as volunteering, for people to participate in their local communities to improve social capital and community engagement. The Marmot Review (2010) [16], provides an evidence-based strategy aiming to tackle the wider social determinants of known health inequalities. Interventions promoting community participation (which might include volunteering) and reducing social isolation were advocated as a means of improving individuals’ health and wellbeing. Furthermore, the US Corporation for National and Community Service (CNCS) released its Strategic Plan for 2011–2015 [7] which focused on increasing the impact of national service on community needs, supporting not only the volunteers’ wellbeing, but prioritising recruitment and engagement of underrepresented populations.

Research into the possible health benefits of volunteering has proliferated recently. A narrative evidence synthesis [10] found volunteering was associated with: increased longevity; improved ability to carry out activities of daily living; better health coping mechanisms; adoption of healthy lifestyles; and improved quality of life, social support, interaction, and self-esteem. Reductions in depression, stress, hospitalisation, pain and psychological distress in volunteers were also reported. However, no experimental studies were identified and the causal effect of volunteering on health remained unclear. Two recent systematic reviews reported narrative evidence of the potential health benefits from volunteering, but findings were restricted to older adults and evidence from only one trial [11,12].

The proliferation of new experimental research, alongside the increased policy focus on volunteering worldwide [7,13,14], provides a timely opportunity to conduct a systematic review and meta-analysis of experimental and longitudinal observational studies of the health effects of volunteering in the general adult population.

The primary aim was to update previous reviews by examining the impact of ‘formal’ volunteering [2] on volunteers’ physical and mental health compared with those individuals who do not volunteer. Secondary aims explored the influence of volunteering type (activity, setting) and intensity on the health benefits observed.

## Methods

This review followed a pre-defined protocol (http://clahrc-peninsula.nihr.ac.uk/includes/site/files/files/EST%20Docs/PenCLAHRC%20volunteering%20protocol%20(21_02_12).pdf), in accordance with the general principles published by the NHS Centre for Reviews and Dissemination [17] and PRISMA (Preferred Reporting Items for Systematic reviews and Meta-Analyses) [18]. Ethical approval was not required.

### Search strategy

The master search strategy composed of MeSH terms and free text words (Table 1) was applied in January 2013. The search was applied to Medline/OVID SP (1950–Present), and adapted for the Cochrane Database (Issue 1, 2013), NHS Economic Evaluation Database (Issue 1, 2013), Embase/OVID SP (1980–2013), PsycINFO/OVIDSP (1987–2013), CINAHL (1981–2013), ERIC (1966–2013), HMIC (1983–2013), Social Science Citation Index (1972–2013), ASSIA (1987–2013), Social Care Online (1980–2013) and Social Policy and Practice (1981–2013). Studies were identified without language, country or date restrictions, by searching electronic databases and scanning reference lists. Non-English language papers were translated.

**Table 1:** Master search strategy

**Search step**	**Database: Ovid MEDLINE(R) in-process &other non-indexed citations and Ovid MEDLINE(R) <1948 to present>**
1	exp voluntary workers/
2	(volunteer* or (charity adj worker*) or (voluntary adj3 worker*) or (voluntary adj group*) or (unpaid adj worker*) or (self-help adj group*)).ti,ab.
3	socially-productive.ti,ab.
4	or/1-3
5	(volunteerism or volunteering).ti,ab.
6	(volunteer adj (work or program* or service*)).ti,ab.
7	((voluntary or charit* or unpaid) adj work).ti,ab.
8	(altruis* or activis*).ti,ab.
9	(self-help or (peer adj support)).ti,ab.
10	(intergenerational adj program*).ti,ab.
11	(community adj (involvement or work)).ti,ab.
12	(helping adj others).ti,ab.
13	(social adj productiv*).ti,ab.
14	(caring or caregiving).ti,ab.
15	(social adj capital).ti,ab.
16	(productiv* adj/2 activit*).ti,ab
17	((civic or community) adj engagement).ti,ab
18	or/5-17
19	(health adj (benefit* or impact* or improvement*)).ti,ab.
20	((impact* or benefit* or improve*) adj2 health).ti,ab.
21	(well-being or wellbeing).ti,ab.
22	“quality of life”.ti,ab.
23	(psychological adj (health or functioning or effect*)).ti,ab.
24	(happiness or satisfaction or rehabilitation or self-esteem or empowerment).ti,ab. (
25	“sense of community”.ti,ab.
26	(community adj connection*).ti,ab.
27	((positive or negative) adj impact*).ti,ab.
28	mortality.ti,ab.
29	or/19-28
30	4 and 18 and 29
31	(volunteering or volunteerism).ti
32	(voluntary adj work*).ti
33	30 or 31 or 32

* Represents a wildcard search term.

Studies were included if they compared the effects of volunteering (with no volunteering) across time on the physical and/or mental health of adults aged 16 years and above. The UN definition of volunteering [1] was adopted. The activity had to be organised in a structured way (‘formal’ volunteering [2]) and take place as a regular, long-term commitment. As there is no agreed definition of what constitutes a ‘long-term commitment’, this review defined this as a minimum of one hour a month on at least two occasions in order for volunteering to impart a plausible and sustained impact. Any reimbursement of expenses had to be considerably less than the commercial value of the work undertaken. A hierarchy of evidence approach [17] was applied; only experimental (randomised and non-randomised controlled trials) and cohort studies were included. Although interpreting evidence of effectiveness from longitudinal analysis of cohort studies may be problematic, they were included due to the limited evidence from experimental studies.

Studies were excluded if: the volunteering activity was a family caring role, spontaneous, unplanned, overseas (e.g. Voluntary Services Overseas or voluntary ‘working’ holidays) or one-off events; the comparator was ‘low level’ volunteering (i.e. less than one hour on two occasions per month); and if the health outcome was not reported at participant-level.

### Screening, data extraction and quality appraisal

One reviewer (CJ) screened all titles and abstracts for eligibility. Uncertainties were checked by a second reviewer (AD) with discrepancies resolved by discussion (CJ, AD and SR). The full text of potentially relevant articles was screened independently by two reviewers (CJ and SR or KJ); discrepancies were resolved by discussion with a third reviewer (SR or KJ). Data were extracted by one reviewer (CJ) and independently checked by a second (KJ or SR) using a standardised, piloted data extraction form.

The methodological quality of each paper was appraised to generate a risk of bias score. These numerical scores were used to aid interpretation only and not as a quality filter when synthesising results. The Cochrane risk of bias tool [19] was used to assess the quality of RCTs. Non-representative samples and high loss to follow-up rates were considered major threats to the external validity of community-based studies. Thus, two domains, random sequence generation and incomplete outcome data, were prioritised out of a possible seven domains. An overall risk of bias score was generated for each study using these prioritised domains.

The Newcastle-Ottawa Scale (NOS) for cohort studies [20] was used to assess the quality of non-RCTs and cohort studies. The NOS allocates points for three domains: selection; comparability; and outcome (maximum score is nine). Part 4 of the ‘Selection’ section (the outcome of interest present at baseline) was excluded as it was deemed not applicable. The risk of bias was then categorised as ‘high’ (0 to 3 points), ‘moderate’ (4 or 5) or ‘low’ (6 to 8).

Health outcomes which included mortality, and physical or mental health measures could be self-reported or extracted from routine records; no quality filtering was applied although the validity of each measure was noted. For physical and mental health measures the following were extracted: the numbers contributing to analysis; the adjusted estimate of between-group effect size (i.e. mean difference or risk ratios); and associated measure of variance (SD or SE) and p-values. Where such data were not available, measures of exposure and outcome association (e.g. correlation coefficients) and associated p-values were extracted.

Many cohort studies analysed facets of the volunteering role as secondary comparisons. The impact on outcomes was extracted for volunteering hours, frequency, the number of organisations supported, the consistency of volunteering (e.g. sustained versus intermittent), the type of activity and the volunteer’s age (older versus younger adults).

### Evidence synthesis

Given the statistical and methodological heterogeneity of studies, meta-analyses of health outcomes were not possible. Consistent with other published reviews [21,22], a more qualitative ‘vote counting’ [23] approach was undertaken. For each study, outcomes were categorised on the basis of statistical significance (p ≤ 0.05) and the direction of effect. To avoid double counting, where multiple papers from the same population cohort reported the same outcome, only one vote per cohort was counted.

As mortality was consistently reported in cohort studies, the mortality risk ratio (relative risk or OR) and 95% CI were extracted. Data were pooled using the STATA (version 12) ‘metan’ command, specifying a random effect meta-analysis model using the DerSimonian Laird method [24]. The I^2^ statistic was used to quantify statistical heterogeneity across cohorts.

## Results

**Figure 1:**
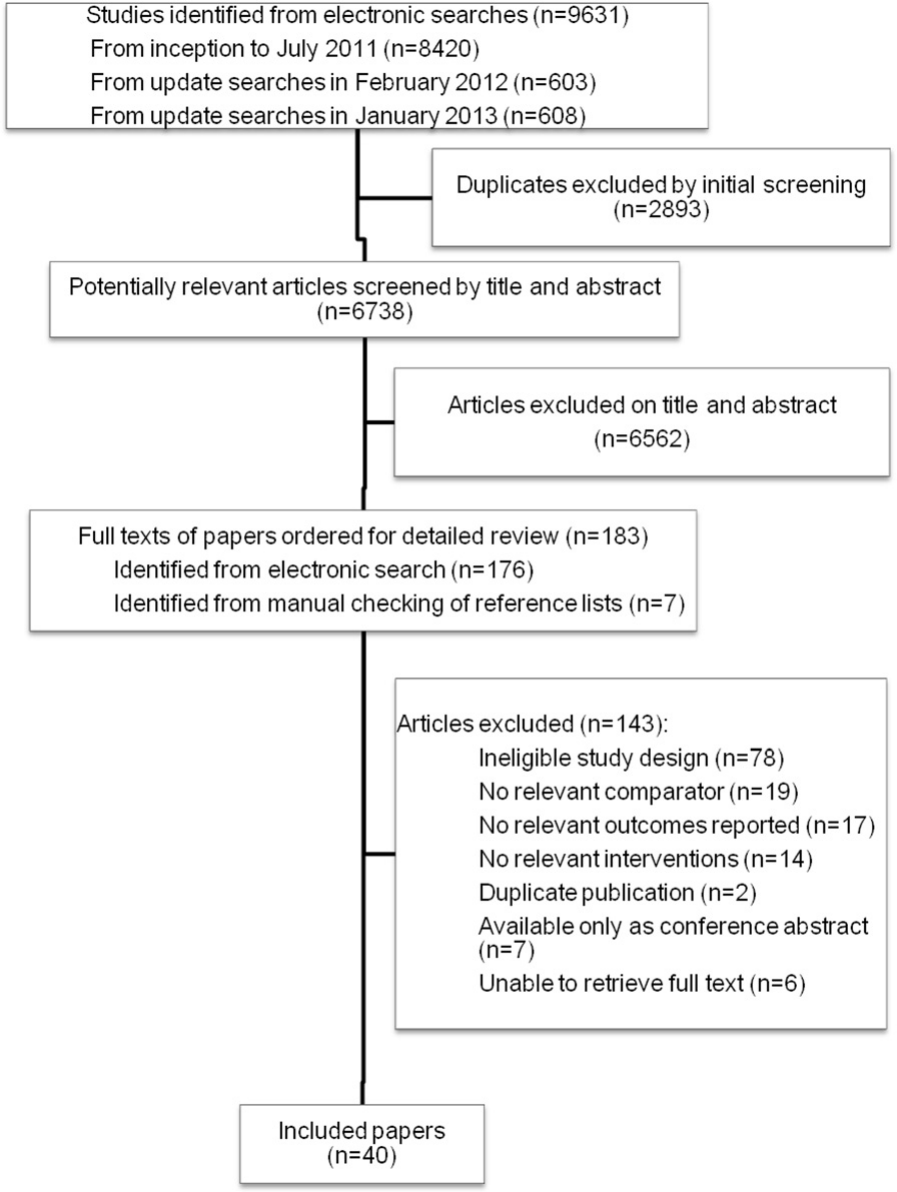
Flow chart of study selection process.

The searches retrieved 9631 papers and a further seven papers were identified through citation tracking. Figure 1 summarises the selection process. Forty papers were included in the review [9,25-63].

### Study characteristics

Eleven papers reported RCTs (n = 7) [30,33,35,37,54,58,62] and non-RCTs (n = 4) [27,29,34,57]. As three RCT papers reported different outcomes collected at the same follow-up from the Experience Corps trial [30,35,58]; a total of five independent RCTs [33,37,54,62] were identified. Of the non-RCTs [27,29,34,57], one paper was drawn from a small sample (n = 18) of Experience Corps trial participants [29] completing a comprehensive battery of cognitive tests that were not routinely administered in the wider cohort. A second paper [57], reported three year follow-up data for a sample of African-American women recruited to the Experience Corps over a two year period compared with matched controls selected from another cohort study.

Twenty-nine papers reported longitudinal analysis of cohort studies; 13 papers reported unique cohorts. The remaining papers reported data from four cohorts: American Changing Lives study (ACL, n = 8) [40-42,46-48,59,60]; the National Survey of Midlife Development in the US (MIDUS, n = 3) [25,31,36]; the Survey of Health, Ageing and Retirement in Europe (SHARE, n = 2) [56,61]; and the Wisconsin Longitudinal Study (WLS, n = 3) [9,51,52].

An in-depth description of each paper (including study design, population, comparator groups, health outcome measured etc.) is summarised in Additional file 1: Table S1 and Additional file 2: Table S2.

### Methodological quality

Four out of five RCTs were at moderate or high risk of bias due to the random sequence generation not being described, high attrition rates, and small sample sizes available for analysis (see Additional file 3: Table S3). Quality appraisal of non-RCTs (see Additional file 4: Table S4) found three non-RCTs were at moderate risk of bias, and one non-RCT at low risk. In contrast, most papers reporting cohort studies (see Additional file 4: Table S4) were large and well designed (25/29 low risk, 4/29 moderate risk).

### Study participants and interventions

#### ***Experimental studies***

All studies were based in the USA and recruited people aged 50 years or over except one Israeli study involving people aged between 19–60 years [33] (Additional file 1: Table S1). The study populations were predominantly female. In the final analyses, there were 308 participants from the five RCTs and 307 participants from the four non-RCTs.

Four RCTs [30,35,37,54,58,62] and one non-RCT [27] investigated intergenerational volunteering interventions; settings included schools [30,35,37,58], a state hospital [54], a long-term care living facility [62] and a retirement facility [27].

The frequency of volunteering varied from 30 minutes to 15 hours a week. Study duration ranged from five weeks to eight months apart from two studies [34,57] that followed-up participants for two to three years.

#### ***Cohort studies***

Most cohort studies (see Additional file 2: Table S2) recruited large samples of community-dwelling adults; only six papers [28,31,36,41,45,49] reported sample sizes of less than 1000 participants. Just one study’s sample included participants from community and institutional settings [44]. Most cohorts (13/17) were in North America, with the remainder located in Israel [26], Germany [43], England [63] and a European collaboration (SHARE) of ten [61] or thirteen nations [56]. Although some cohorts (e.g. ACL, MIDUS, WLS) recruited adults of all ages, most papers restricted analysis to participants aged 50 years or over.

The proportion of participants who reported volunteering varied considerably (5.7% [55]-75.6% [45]). Direct comparisons both between- and within-cohorts were problematic due to differences in samples and definition of volunteering status. Some papers describing complex multivariate analyses omitted basic descriptive information on the prevalence of volunteering [9,28,41,42,51,52,63]. Notwithstanding this, volunteering estimates derived from Japan (5.7% [55]), Israel (10.7% [26]), Europe (12.4% [56], 15.41% [61]) and Germany (23% [43]) were generally lower than those from North America; here only 3/17 papers reported volunteering rates below 30% [38,39,44]. Participants’ age appeared to influence prevalence rates; studies using baseline samples of predominantly younger adults yielded higher estimates of volunteering rates [40,45,53,60] than those composed mainly of older adults.

Descriptive data (see Additional file 2: Table S2) on the nature (e.g. setting, type of activity, frequency or duration) of the volunteering activities were relatively sparse and no clear patterns emerged. ollow-up length varied considerably between cohorts and papers e.g. 1–10 years (12 cohorts, 22 papers) [9,25,26,31,32,36,38-42,46-50],[55,56,59-61,63], and 14–30 years (5 cohorts, 7 papers) [28,43-45,51-53].

### Impact of volunteering on health outcomes and survival

#### ***Experimental studies***

Additional file 5: Table S5 summarises the impact of volunteering on physical and mental health outcomes. Only outcomes relating to depression, self-rated health, self-esteem and cognitive function were reported by more than one trial.

Vote counting did not find any consistent, significant health benefits arising through volunteering. Three RCTs found no between-group differences in depression [37,54,62], one RCT [54] and two non-RCTs [27,34] found no significant differences in self-esteem, and an RCT [62] and one non-RCT [34] found no difference in self-rated health. Measures of cognitive function varied within the Experience Corps trial [29,30,35] and another RCT [37]. Only one RCT [29] found volunteering significantly improved cognitive function. Two trials reporting data on purpose in life found no significant effect [34,37].

All other health outcomes were only measured by one trial, with volunteering significantly associated with increased physical activity [35,57,58], strength [35], walking speed [35], empowerment [33], wellbeing [62], and decreased stress [37]. No significant effects were found for the number of falls in the previous year [35], cane use [35], sense of usefulness [37], and loneliness [54].

#### ***Cohort studies***

Additional file 6: Table S6 summarises the impact of volunteering on survival, and physical and mental health outcomes reported in cohort studies.

Survival rates were reported in seven studies [9,26,38,44,47,49,50], with most follow-ups ranging from 4–8 years; only one study followed participants for 25 years [44]. Three studies reported no association with volunteering [9,44,49]. The remainder found statistically significant associations between at least one measure of volunteering status or intensity (e.g. frequency, hours spent, number of organisations supported) and mortality [26,38,47,50]. Interpretation was difficult, however, as studies reported statistically significant, but contradictory associations.

**Figure 2:**
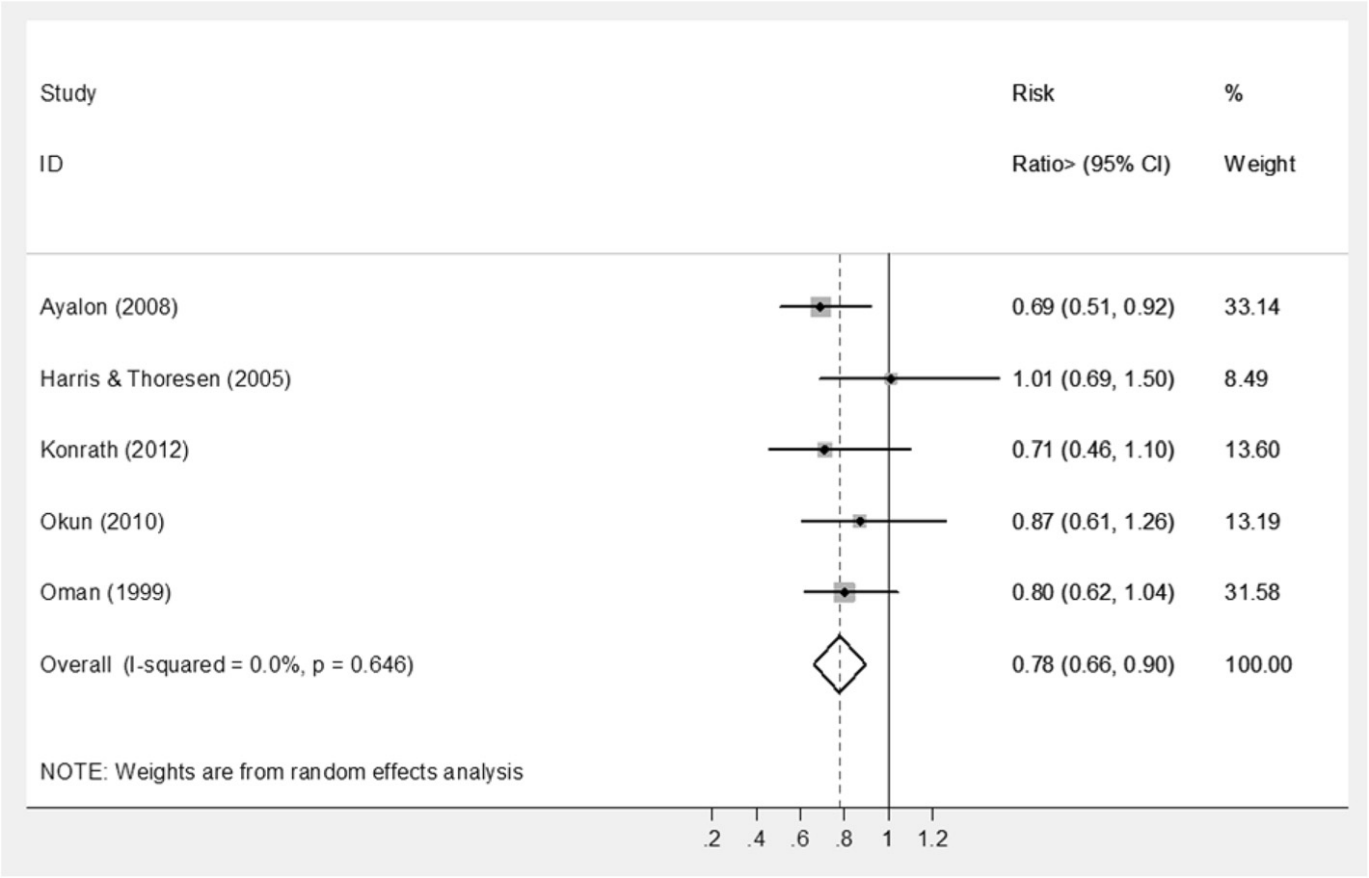
Forest plot to illustrate the effect of volunteering on risk of mortality.

Sufficient data were available to pool mortality data for five studies [9,26,38,49,50] with participant follow-ups ranging from four to seven years. After adjusting for important potential socio-demographic and health-related confounders, volunteers had a significantly lower risk of mortality (risk ratio: 0.78; 95% CI: 0.66, 0.90; I^2^ test: p = 0.65) compared to non-volunteers (Figure 2).

Vote counting was possible for physical functional abilities and self-rated health. Three cohorts [42,45,46,55,59] reporting functional abilities (activities of daily living) yielded inconclusive evidence, partially due to the way volunteering was measured. The ACL cohort found both volunteering status and the number of hours spent volunteering improved functional dependency [46,59]. However, more sophisticated path analysis suggested a clustering effect in people aged 60 years and above compared to their younger counterparts [42]. In contrast, no relationship was found between functional ability and volunteering status [55]. The remaining study [45], reported that intermittent volunteering (as opposed to sustained participation) resulted in benefits, while neither the age nor transitions (i.e. starting/stopping volunteering) impacted on functional ability outcomes.

Four cohorts [45,46,52,53,59,60] reported self-rated health, three of which assessed outcomes for 20 years or more [45,52,53]. Volunteering status was associated with higher levels of self-rated health in two cohorts [46,52,59,60]. A third study [53] found benefits were associated with environmental volunteering rather than civic volunteering or no volunteering, while another [45] reported no benefits.

Single papers reported other physical health outcomes. Environmental volunteering was associated with higher levels of physical activity across a 20 year follow-up compared with either civic volunteering or no volunteering [53]. Neither volunteering status nor hours spent volunteering were associated with frailty [39] (three year follow-up), and no association in the number of chronic conditions reported [59] was found (eight year follow-up).

Vote counting was possible for depression, life satisfaction, wellbeing, and quality of life. Depressive symptoms were assessed in six cohorts [32,36,40-42,46,48,51,53,63], with follow-ups ranging from 2–20 years. Irrespective of how it was measured, volunteering was associated with reduced levels of depression in four cohorts [32,40-42,46,48,51,63], with two cohorts reporting no benefits [36,53]. Of cohorts reporting benefits, it was difficult to synthesise clear messages as the way volunteering was modelled (status, intensity, consistency etc.) varied considerably. Analyses of the ACL cohort suggested that while volunteering status and the hours spent volunteering were associated with improved outcomes, this benefit may be limited to older volunteers [40,42,48]. Data from ACL [48], English Longitudinal Study of Ageing (ELSA) [63] and WLS [51] cohorts suggested that benefits only accrue through sustained rather than intermittent volunteering.

Four [28,43,60,63] of the five cohorts [28,43,44,60,63] that assessed life satisfaction reported benefit (3–25 year follow-ups). Two studies explored the effect of volunteering intensity, with improvements being associated with greater time spent (hours) [60] and/or a regular, weekly commitment [43]. One study found benefits were associated with sustained rather than intermittent volunteering [63].

Volunteering status was significantly associated with improved wellbeing in three cohorts [25,28,31,51,52] (10–29 year follow-ups) but findings regarding volunteering intensity were inconsistent. WLS data [51,52] suggested that greater benefits were associated with sustained or intermittent volunteering, and volunteering for a diverse range of organisations whereas MIDUS data [25,31] found benefits only accrued with volunteering one to ten hours per month (with no associated benefit with greater time commitments).

Improved quality of life was associated with volunteering status in two cohorts [56,61,63] (2–5 year follow-ups) but only if the activity was reciprocal, i.e. the volunteer felt their actions to be appreciated [56,63]. Some mental health outcomes were only analysed in one cohort. Volunteering was associated with improved self-efficacy for activities of daily living (one year follow-up) [55] but not with ‘happiness’ (25 year follow-up) [44].

## Discussion

### Interpreting study findings

This systematic review and meta-analysis has updated the evidence base regarding the potential health benefits of volunteering. By removing adult age and language filters, trials and cohort studies deemed ineligible by earlier reviews [11,12] were included. Furthermore, volunteering interventions were systematically described and the impact on health outcomes of factors such as volunteering intensity and duration, and volunteers’ characteristics (e.g. age, gender) were summarised.

Heterogeneous findings were observed in the five trials [30,35,37,54,58,62] investigating the health effects of intergenerational volunteering among older adults, with benefits reported for some elements of physical activity and cognitive function. No significant effects were observed for depression, self-rated health or self-esteem. However, all studies recruited small samples that were likely to be underpowered to detect important between-group differences, and this was exacerbated by sample attrition.

Most cohort studies recruited large samples with lengthy follow-ups, thus being at low risk of bias. Meta-analysis of five studies [9,26,38,49,50] identified a 22% reduction (CI: 10% to 34%) in mortality among volunteers compared to non-volunteers. Vote counting failed to identify any consistent beneficial effects of volunteering on either physical functional ability or self-rated health. For mental health, volunteering had a favourable effect on depression, life satisfaction and wellbeing. With the possible exception of wellbeing [62], the limited trial evidence did not support findings from observational studies.

Conflicting results from studies exploring the influence of volunteering type and intensity on the magnitude of observed health benefits prevented any clear evidence being synthesised.

Several limitations should be acknowledged. While meta-analysis of survival data was undertaken, analysis of the remaining physical and mental health outcomes was restricted to vote counting [23] due to heterogeneous trial interventions and study methods of both trial and observational studies. The generalisability of the evidence reviewed here is also limited. Indeed, most studies were based in the USA where there is a strong history of volunteering and a wide disparity in health, and involved samples of community dwelling people aged 50 years or over. The relevance of the current findings on a nation where health inequalities and volunteering are less prevalent may be questionable. Unfortunately, many studies based outside the USA reported cross-sectional data that were excluded at the study eligibility stage of the review. Reassuringly, the estimates of the prevalence of volunteering from observational studies is consistent with other sources [3,5], which found the prevalence of volunteering is generally higher in the USA compared with European cohorts, and that older people may be less likely to volunteer than their younger counterparts [64].

A key challenge remains in unpacking the theoretical mechanisms by which volunteers accrue specific health benefits. This poses an interesting hypothesis that different health benefits are accrued in different and potentially antagonistic ways. For example, the tentative effect of volunteering on physical activity [35,53,57,58] may simply be explained by the increase in the number of trips out of the house, for whatever reason [65,66]. Here, in terms of dosage, more volunteering would have greater effects on physical activity and associated physical health outcomes. However, it emerges the opposite may be true for mental health; i.e. less volunteering may be more beneficial. Although people tend to volunteer for altruistic reasons [9], if reciprocity is not experienced, then the positive impact of volunteering on quality of life is negated [56,63]. The importance of reciprocity was highlighted in the cross sectional analysis of the English Longitudinal Study of Ageing (ELSA); retired people who engaged in either paid work or volunteering experienced greater levels of wellbeing compared to those retirees who engaged in caring [8]. Similar trends were found in employed and/or volunteering older caregivers (aged 60 years or above) who reported better self-rated health compared to those older caregivers who did neither activity [67]. However, there may be a fine line between volunteering enough to experience mental health benefits (e.g. up to ten hours a month) and spending too much time volunteering so that it becomes another commitment [31]. If volunteering becomes a burden, this may lead to ‘burnout’ and possibly giving up volunteering [9,54]. An individual’s life history also influences the impact of volunteering. The small number of observational studies that stratified analysis by age found that older people may be more likely to experience reduced functional dependency and fewer depressive symptoms through volunteering compared with their younger counterparts [40,42,48], although one study found no such benefit [45].

Another key challenge is to explain why volunteering has such a significant impact on survival given the lack of robust changes in physical and mental health outcomes. Selection effects driven by unknown confounders cannot be conclusively ruled out when interpreting the survival data from observational studies. Similarly reverse causality cannot be completely discounted as the volunteers are often from more affluent backgrounds and in better health than non-volunteers [6,8,63]. To limit such effects, meta-analysis pooled mortality risk data after adjustment for baseline between-group differences in socio-demographic, economic, lifestyle and physical and mental health status. While such adjustments strongly mediated survival, a significant effect remained. Social integration also mediated the relationship between survival and volunteering status [38,50]. Since people reporting stronger social relationships have a reduced risk of mortality [68], the social aspects of volunteering may contribute to the observed survival differences. Taken together, this review suggests that bio-social and cultural factors may influence both a willingness to engage in volunteering, as well as the benefits that might accrue.

This review aimed to identify evidence regarding the health benefits of formal volunteering undertaken on a sustained and regular basis. Although unproblematic when considering trial eligibility, many cohort studies failed to fully describe how volunteering status was defined or measured. This is unsurprising given the nature of these large, population cohort studies; volunteering is often only one of many social activities assessed. An inclusive approach was adopted to maximise the evidence available. Tighter study inclusion criteria would not only result in many observational studies being omitted from this analysis, but might substantially change the findings.

### Implications for health inequalities, practice and research

The State of the World’s Volunteerism Report 2011 [14], the Policy Agenda for Volunteering in Europe (PAVE) [13], the CNCS Strategic Plan 2011–2015 [7] and the UK government policy [15] advocate the uptake of volunteering as a method of improving civic engagement, with the added potential of improving participants’ health and wellbeing [16]. Alongside more traditional health promotion goals, such as reducing physical inactivity and excess weight in adults, the new Public Health Framework for England (2013–16) [69] includes self-reported wellbeing and improving health-related quality of life for older people as indicators. In this review, the potential for advocating volunteering as a public health promotion intervention to improve physical and mental health outcomes was explored.

Many uncertainties remain that preclude clear recommendations for practice. For example, it is unclear what type or dose of volunteering activity is associated with the greatest health improvement, for which outcomes and for whom. While the underlying causal mechanisms cannot be explained due to the potential for reverse causation and selection bias, synthesis of observational data suggests that people who choose to volunteer are at a lower risk of mortality, and may experience some benefits in terms of physical and mental health. With the lack of experimental evidence, this could be interpreted as proof of no public health benefits arising through volunteering roles. However, given the methodological limitations of trial evidence (e.g. small selected sample sizes), it must equally be acknowledged that such evidence cannot conclusively rule out the potential for volunteering as a public health intervention. If it is accepted that volunteering may result in health benefits, perhaps the key challenge to practitioners is how to achieve wider participation amongst socially-disadvantaged groups [7,8,35,57] at the greatest risk of experiencing health inequalities. Socially-inclusive volunteering interventions, such as the Experience Corps Program [35], require careful planning and partnership working with the voluntary sector, to ensure that barriers to participation for disadvantaged groups are identified and removed. While having the potential to be a low cost, sustainable intervention, service commissioners must recognise that the infrastructure required to improve community engagement is not cost free.

## Conclusions

Future research is urgently needed to explore the underlying causal mechanisms between volunteering and mortality. This review has highlighted the need for a deeper understanding into the delivery of volunteering (e.g. frequency, dose, type of activity) required to yield optimal health benefits. Furthermore, it is essential to measure a health outcome that would plausibly be affected by the volunteering intervention (e.g. measuring physical activity while undertaking environmental volunteering as opposed to listening to children read). Analysis should also focus on the impact of potential mediating factors associated with the promotion of healthy lifestyles (e.g. physical activity, physical functioning), mental wellbeing (e.g. stress reduction, affective states), and social participation. Documenting the degree to which motivating and sustaining factors, such as altruism [9] and reciprocity [56,63] are core components of this complex intervention is critical, as volunteering interventions are unlikely to yield benefits if such activities hold no intrinsic meaning or value to the potential recipients. However, ‘volunteering’ (as assessed in cohort studies) is rarely described and heterogeneous in nature suggesting that future evaluations must seek to better describe the intervention ‘tested’. This raises the possibility that the very definition of volunteering, as an act of free will and choice, is essentially incompatible with the notion of a (randomised) intervention and evaluation [10]. The emerging science of evaluating the impact of complex health behavioural change is giving new insights into intervention mapping and development [70]. By adopting this approach, it may be more feasible over the long-term, to robustly design and adequately power, pragmatic RCTs. Crucially, such interventions need to engage with and recruit from socially diverse communities in order to test the effectiveness of volunteering as a public health intervention.

## Competing interests

The authors declare they have no competing interests.

## Authors’ contributions

SR, CJ, AD, JTC and IL conceived and designed the review. All authors interpreted the data, critically revised the manuscript for important intellectual content and approved the final versions. In addition, CJ, SR and AD undertook the pilot work, screened titles, abstracts and full texts and applied inclusion and exclusion criteria. CJ performed data extraction and quality appraisal and KJ checked data extraction. CJ, SR and JTC drafted the manuscript. RT provided statistical support. MR devised the search strategy and ran the literature searches. SR is the guarantor.

## Pre-publication history

The pre-publication history for this paper can be accessed here:

http://www.biomedcentral.com/1471-2458/13/773/prepub

## Supplementary Material

Additional file 1: Table S1 Characteristics of experimental studies (9 trials, 11 papers). Click here for file

Additional file 2: Table S2 Characteristics of longitudinal cohort studies (17 unique cohorts, 29 papers). Click here for file

Additional file 3: Table S3 The Cochrane Collaboration’s tool for assessing risk of bias (5 trials, 7 papers). Click here for file

Additional file 4: Table S4 NOS scores of non-RCTs and longitudinal cohort studies (4 non-RCTs, 17 unique cohorts, 33 papers). Click here for file

Additional file 5: Table S5 Vote counting for experimental study designs (9 trials, 11 papers). Click here for file

Additional file 6: Table S6 Vote counting for longitudinal study designs (17 unique cohorts, 29 papers). Click here for file
