# Supplementary material for: Is volunteering a public health intervention? A systematic review and meta-analysis of the health and survival of volunteers
Source: BMC Public Health. 2013 Aug 23;13:773. doi: 10.1186/1471-2458-13-773 (PMC3766013; doi:10.1186/1471-2458-13-773)
Supplement: Additional file 1: Table S1 — Characteristics of experimental studies (9 trials, 11 papers). [file 1471-2458-13-773-S1.docx]

**Table S1 Characteristics of experimental studies (9 trials, 11 papers)**

| **Author, year (country)** | **Subject population and cohort (if relevant)** | **Intervention** | **Comparator** | **Number of follow-up time points (schedule)** | **Outcomes considered** |
| --- | --- | --- | --- | --- | --- |
| RCT (5 trials, 7 papers) | | | | | |
| Carlson et al, 2008;  Fried et al, 2004;  Tan et al, 2006  (USA) | Community-dwelling (≥60 years)  Experience Corps Program | Setting: elementary school  Activity: one-on-one or small group literacy support, library support, violence prevention activities, attendance enhancement and/or other formal roles in which they were trained  Frequency: ≥15 hours a week (over 3-5 days)  Duration: for a complete school year | Waiting list | 2 (baseline; 4-8 months depending on enrolment date) | Mental health ­(Carlson 2008)  Cognitive function:  Executive function (Trail Making Test)  Memory (Rey-Osterreith Complex Figure Test)  Verbal learning  Physical health (Fried 2004)  Physical activity  Strength  Number of falls  Cane use  Walking speed  Mental health  Cognitive function  Physical health (Tan 2006)  Physical activity (MLTPAQ and 3 items from Paffenbarger) |
| Cohen, 2009 (Israel) | Social Services clients (19-60 years) | Setting: various depending on activity  Activity: visiting elderly, manning hotline, instrumental assistance and enrichments in day care centres for the elderly, mentoring for children at risk, distribution of food in soup kitchens, socialising in a disability centre, providing instruments and socialising in kindergartens  Frequency: 2 hours a week  Duration: 6 months | Setting: social services department  Activity: supervision, attend volunteer parties and trips  Frequency: not reported  Duration: 6 months | 2 (baseline; 6 months) | Mental health  Empowerment (composites of cognitive elements, critical awareness, self-efficacy, interpersonal and political skills) |
| George & Singer, 2011  (USA) | Residents of an assisted living facility (>50 years, diagnosis of mild to moderate dementia) | Setting: intergenerational school  Activity: alternate weeks, participants served as mentors in a kindergarten class (5-6 years old), interacted with children and engaged in singing and small group reading and writing activities. In a 6th grade class (11-14 years old) intergenerational life-history reminiscence sessions with smaller groups of 2-3 students  Frequency: 1 hour a week  Duration: 5 months (total output of 20 hours) | Setting: Judson Park (assisted living facility)  Activity: a peer educational seminar called "Successful Aging: Reclaiming Elderhood", 8 homework assignments between sessions that were intended to take 1hour each to complete  Frequency: 8 seminars totalling 12 hours  Duration: 5 months (the output of hours match those of the intervention group) | 2 (baseline; 5 months) | Mental health  Cognitive function (MMSE)  Anxiety (BAI)  Depression (BDI)  Sense of purpose and sense of usefulness (single-item questionnaire) |
| Rook & Sorkin, 2003^a^(USA) | Community-dwelling (≥60 years)  Foster Grandparent Program (FGP) | Setting: state hospital  Activity: each FGP participant was assigned a developmentally-disabled child ("client") in residence at the hospital e.g. spending time with the client, taking client on excursions available on the hospital grounds, transporting client to on-site clinics and Drs appointments  Frequency: 4 hours per day, 5 mornings each week  Duration: 2 years | Setting: regional nutritional centres  Activity: a non-volunteer, alternative group program (AGP) that offered access to age peers, meals, $50 monthly stipend  Frequency: not reported  Duration: 2 years  The Community Sample was not randomly selected as part of the FGP so this data was excluded from all our data synthesis. | 3 (baseline; annually for 2 year period) | Mental health  Self-esteem (10 item Rosenberg)  Loneliness (10 item UCLA scale)  Depression (20 item CES-D) |

| Yuen et al, 2008 (USA) | Residents of long term care (LTC) facilities (≥60 years) | Setting: LTC facility  Activity: one-to-one basis, each resident was paired with an English as a Second Language student to help conversation skills, pronunciation of words, meaning of American expressions and correct use of words in different contexts  Frequency: 1hour, twice a week  Duration: 12 weeks but does not state how long each session lasted | Setting: LTC facility  Activity: usual customary social and recreational activities available at the facility (did not involve voluntary work)  Frequency: not reported  Duration: 12 weeks | 3 (baseline; post intervention (12 weeks); 3 months) | Physical health  Self-rated health  Mental health  Depression (GDS)  Life satisfaction (LSI-A)  Data analysis pooled these outcomes to give an overall wellbeing measure |
| --- | --- | --- | --- | --- | --- |
| Non-RCTs (4 trials) | | | | | |
| Belgrave, 2011 (USA) | Residents of a retirement living facility  Unique | Setting: a retirement living facility  Activity: ten 30 minutes music therapy intergenerational sessions  Frequency: ten 30 minute sessions  Duration: 15 weeks | Setting: a retirement living facility  Activity: usual activity programs  Frequency: not reported  Duration: 15 weeks | 2 (baseline; F1 15 weeks) | Mental health  Self-esteem (Rosenberg) |
| Dabelko-Schoeny et al, 2010 (USA) | Attendees of two Adult Day Health Service (ADS) programmes (≥60 years) | Setting: ADS centre  Activity: 5-session multicomponent: education, service and recognition. Education phase: participants learned about community they would serve. Service phase: assembling care packages for community groups (organising purchased or donated items, placing into bags and writing or dictating personal notes to recipients). Recognition phase: participants presented the completed package to a representative of each community group who personally thanked them for their contribution.  Frequency: not reported  Duration: 5 weeks | Setting: ADS centre  Activity: usual activity programs e.g. arts and crafts, physical activity, and intellectual stimulation including discussing current events  Frequency: not reported  Duration: 5 weeks | 2 (baseline; time of switch, 5 weeks)  This study was a non-equivalent switching replications design but for the purpose of the review, only the first half of the design was analysed. | Physical health  Self-rated health  Mental health  Purpose in life (subscale from Ryff)  Self-esteem (10 item Rosenberg) |
| Carlson et al, 2009^b^  (USA) | Community-dwelling African American women (≥60 years) from the Experience Corps Program | See Carlson et al, 2008 | Waiting list | 2 (baseline; 6 months) | Mental health  Cognitive function (Flanker test, and functional MRI data of the anterior cingulate cortex, left and right dorsal prefrontal cortex, and left and right ventral prefrontal cortex) |
| Tan et al, 2009^b^ (USA) | Community-dwelling African American women (≥65 years) from the Experience Corps Program  Community dwelling African American women (aged 65-86 years)  Baltimore Women's Health and Aging Study (WHAS) cohorts I & II | See Carlson et al, 2008  Duration: 3 years  (Data imply that the EC participants volunteered for the full 3 years but the cross referenced data reported 20% of volunteers completing the first year left the intervention (Fried et al, 2004). It is also unclear if participants in the waiting list control group on the RCT subsequently became part of this current dataset.) | No intervention: WHAS I & II are longitudinal observational cohort studies of older women | 4 (baseline; 12; 24; and 36 months) | Physical health  Physical activity (modified MLTPAQ) |

^a^The community sample arm of the trial which was not part of the randomisation process was ignored.

^b^ These two papers report very different experiments using Experience Corp participants and are therefore presented separately.

BAI, Beck Anxiety Index; BDI, Beck Depression Index; CES-D, Center Epidemiological Studies-Depression; GDS, Geriatric Depression Scale; LSI-A, Life Satisfaction Index-A; MLTPAQ, Minnesota Leisure Time and Physical Activity Questionnaire; MMSE, Mini-Mental State Examination; Rosenberg, Rosenberg Self-Esteem Scale; Ryff, Ryff Psychological Wellbeing Scale; UCLA, University of California, Los Angeles Loneliness Scale
