# Supplementary material for: Is volunteering a public health intervention? A systematic review and meta-analysis of the health and survival of volunteers
Source: BMC Public Health. 2013 Aug 23;13:773. doi: 10.1186/1471-2458-13-773 (PMC3766013; doi:10.1186/1471-2458-13-773)
Supplement: Additional file 2: Table S2 — Characteristics of longitudinal cohort studies (17 unique cohorts, 29 papers). [file 1471-2458-13-773-S2.docx]

**Table S2 Characteristics of longitudinal cohort studies (17 unique cohorts, 29 papers)**

| **Authors, year (country)** | **Subject population and cohort (if relevant)** | **Intervention^a^** | **Analyses adjusted for confounders**  **(at baseline unless stated)** | **Number of follow-up time points (schedule: baseline, F=follow-up)**  **Total duration** | **Outcomes** |
| --- | --- | --- | --- | --- | --- |
| Unique cohorts (n=13) | | | | | |
| Ayalon, 2008 (Israel) | Community dwelling (≥60 years)  Unique | Setting: within an organisation or provide ongoing assistance to non-family members  Activity: not described  Frequency: hours a week: 0; <1 hour; 5-9 hours; >10 hours; a varied number of hours  Duration: number of years volunteering: ≤4 years; 5-9 years; 10-14 years; >15 years | age, gender, education, physical and mental health, activity level, social ties | 2 (baseline 1997-8; F1 2004)  7 years | Physical health  Mortality |
| Bowman et al, 2010 (USA) | First year university students in 1990 (17-19 years)  Unique | Setting: various e.g. school or education based, religious or faith-based, advocacy or issue group  Activity: various  Frequency: varies across follow-ups e.g. hours a week, never to frequently  Duration: varies across follow-ups | age, gender, race, degree attainment, marital status, number of children, employment, income, religious engagement, college satisfaction, ill-being at college | 3 (baseline 1990; F1 1994; F2 2007)  17 years | Mental health  Wellbeing (dimensions Ryff)  Life satisfaction (Diener) |
| Choi & Bohman, 2007 (USA) | Community dwelling (≥65 years)  HRS | Setting: not reported  Activity: formal volunteering  Frequency: not reported  Duration: not reported | age, race, education, health/disability CES-D Score, F1 death, F1 loss contact, CES-D score missing, changes in marital status, work, caring status, health/disability, health behaviours since baseline | 2 (baseline 1998; F1 2000)  2 years | Mental health  Depression (8-item CES-D) |
| Harris & Thoresen, 2005 (USA) | Community dwelling (≥70 years)  LSOA | Setting: various  Activity: helping in charity work, working in a shop for non-profit organisation, working in a hospital or nursing home without pay or doing community work without pay  Frequency: never, rarely, sometimes, frequently  Duration: past 12 months | age, gender, ethnicity, education, marital status, income, veteran, number of chronic conditions, health status and limitations, physical activity, social integration, social functioning and support, attends church | 4 (baseline 1984; F1 1986; F2 1988; F3 1990)  6 years | Physical health  Mortality |
| Jung et al, 2010 (USA) | Community dwelling (70-79 years)  MSSA | Setting: not stated but uses ACL measures (e.g. church, synagogue, other religious organisation, school or educational organisation, political group or labour union, senior citizen group or rerated organisation, any other national or local organisation including United Fund, hospitals)  Activity: not reported  Frequency: number of hours (no engagement, ≤39 hours, 40-159 hours, ≥160 hours)  Duration: past 12 months | age, gender, race, education, marital status, medical comorbidity, physical disability, intermediate frailty, cognitive function, depressive mood, personal mastery belief, social club participation, emotional support, religious service attendance , engagement of productive activities, | 2 (baseline 1988; F1 1991)  3 years | Physical health  Frailty |
| Meier and Stutzer, 2008 (Germany, spanning the reunification of East and West Germany) | Community dwelling (adults)  GSOEP | Setting: implies formal settings such as political organisations, old people's home, sport organisation  Activity: not reported  Frequency: weekly, monthly, less frequently, never  Duration: not reported | Age, gender, marital status, children, employment, income, West or East Germany, nationals, feeling insecure | 11 (data collected yearly from 1985-1999 but only 11 waves were used as volunteering was excluded in the years 1987, 1989, 1991 and 1993)  14 years | Mental health  Life satisfaction |

| Menec, 2003 (Canada) | Community and institutional dwelling (≥65 years)  AIM | Setting: not reported  Activity: volunteer work  Frequency: not reported, asked to tick if volunteered  Duration: past week | age, gender, education, activities for daily living, instrumental activities for daily living, physical difficulties, self-rated health, morbidity, cognitive impairment, life satisfaction | 2 (baseline 1990; F1 1996)^b^  25 years | Physical health  Mortality  Function: composite measure based on ADLs, IADLs, MSQ (cognitive impairment), physical difficulties  Mental health  Life satisfaction (LSI-A) Happiness |
| --- | --- | --- | --- | --- | --- |
| Moen et al, 1992 (USA) | Community dwelling wives and mothers in 1956 (adults)  Unique | Setting: implies community based volunteering  Activity: implies formal volunteering  Frequency: number of years  Duration: from the ages 18-55 years | age, education, poor health number of roles, F1: marital status, household composition, number of children, duration of marriage, timing duration and number of spells employment, duration of time caring for preschoolers, duration of time caring for other relatives | 2 (baseline 1956; F1 1986)  30 years | Physical health  Functional ability  Self-rated health |
| Nazroo & Matthews, 2012 (England) | Community dwelling (women ≥60 years, men ≥65 years)  ELSA | Setting: organisation (fundraising, campaigning, committee, helping with an event, admin work) and person-based (visiting/ befriending, education/counselling, helping with household tasks, providing personal care)  Activity: not reported  Frequency: never, less than once a year, about once or twice a year, every few months, once a month, twice a month or more  Duration: past month | age, gender, marital status, self-perceived social status, wealth, self-reported health, a disability score based on Activities of Daily Living and Instrumental Activities of Daily Living, economic activity, involvement in care giving | 3 (baseline 2004-5; F1 2006-7; F2 2008-9)  5 years | Mental health  Depression (8 item CES-D)  Quality of life (CASP-19)  Life satisfaction (5 item Diener) |
| Okun et al, 2010 (USA) | Community dwelling (56-91 years)  LLSSE | Setting: not reported  Activity: not reported  Frequency: never or almost never, once a month or less, several times a month, about once a week, several times a week, daily  Duration: past month | age, gender, education, marital status, employment, self-rated health, number of chronic health conditions, exercise frequency, attend meetings of clubs and organisations, religious services attendance | 2 (baseline 2000; F1 2006)  6 years | Physical health  Mortality |
| Oman et al, 1999 (USA) | Community dwelling (≥55 years)  Unique | Setting: not reported  Activity: formal volunteering  Frequency: hours a week  Duration: “at the present time” | age, gender, ethnicity, education, employment, income, physical health and functioning status, health habits, self-rated health, depression, memory, social functioning and support | 2 (baseline 1990-1; F1 1995)  5 years | Physical health  Mortality |
| Pillemer et al, 2010 (USA) | Community dwelling (adults)  ACS | Setting: a group concerned with children (e.g. PTA, boy scouts); a group concerned with community betterment, charity, or service; a church connected group; a group concerned with public issues such as civil liberties, property rights etc; a group concerned with self improvement that meets regularly; a group concerned with the environment, pollution etc  Activity: not reported  Frequency: very active, somewhat active, inactive  Duration: not reported | age, gender, education, marital status, physical activity, chronic conditions, functional impairment, depression, social isolation | 2 (baseline 1974; F1 1994)  20 years | Physical health  Physical activity  Self-rated health  Mental health  Depression (Kaplan et al) |
| Shimanuki et al, 2007 (Japan) | Community dwelling (70-84 years)  Unique | Setting: long-term care prevention programme  Activity: hold workshops, meetings, mini-day service and classes to prevent falls for the general elderly  Frequency: 2 hours session, 1-4 times a month  Duration: 7 months | age, gender, education | 2 (baseline 2003; F1 2004)  1 year | Physical health  Instrumental independence  Self-efficacy for ADLs |

| ACL (8 papers) | | | | | |
| --- | --- | --- | --- | --- | --- |
| Kim & Pai, 2010 (USA) | Community dwelling (≥25 years)  ACL | Setting: various (e.g. church, synagogue or other religious organisation, school or educational organisation, political group or labour union, senior citizen group or related organisation, any other national or local organisation including United Fund, hospitals)  Activity: not reported  Frequency: number of hours (none, 1-19 hours, 20-39 hours, 40-79 hours, 80-159 hours, ≥160 hours)  Duration: past 12 months | age, gender, race, education, income, functional limitations | 3 (baseline 1986; F1 1989; F2 1994)  8 years | Mental health  Depression (11 item CES-D) |
| Li & Ferraro, 2005 (USA) | Community dwelling (≥60 years)  ACL | See Kim & Pai, 2010  Frequency: number of hours (no engagement; ≤39 hours; 40-159 hours; ≥160 hours) | age, gender, ethnicity, education, income, functional impairment , social integration | 3 (baseline 1986; F1 1989; F2 1994)  8 years | Mental health  Depression (6 item CES-D) |
| Li & Ferraro, 2006 (USA) | Community dwelling (<40 years)  ACL | See Kim & Pai, 2010 | age, gender, race, education, marital status, children, employment, income, chronic conditions, self-esteem, formal meeting attendance, social integration, religious attendance | 3 (baseline 1986; F1 1989; F2 1994)  8 years | Physical health  Functional limitations  Mental health  Depression (6 item CES-D) |
| Morrow-Howell et al, 2003 (USA) | Community dwelling (≥60 years)  ACL | See Kim & Pai, 2010 | F2 age, F2 gender, F2 race, F2 education, F2 marital status, F2 income, F2 social integration, baseline and F1: functional dependency, self-rated health, depression | 3 (baseline 1986; F1 1989; F2 1994)  8 years | Physical health  Functional dependency  Self-rated health  Mental health  Depression (Modified CES-D) |
| Musick & Wilson, 2003 (USA) | Community dwelling (≥25 years)  ACL | See Kim & Pai, 2010 | age, gender, race, education, marital status, employment, income, functional impairment, chronic health problems, physical activity, F1 self-esteem, F1 meeting attendance, F1 social interaction, religious service attendance | 3 (baseline 1986; F1 1989; F2 1994)  8 years | Mental health  Depression (11 item CES-D) |
| Musick et al, 1999 (USA) | Community dwelling (≥60 years)  ACL | See Kim & Pai, 2010 | age, gender, race, education, income, physical activity and health, social integration | 2 (baseline 1986; F1 1994)  8 years | Physical health  Mortality |
| Tang, 2009 (USA) | Community dwelling (≥60 years)  ACL | See Kim & Pai, 2010 | age, gender, race, education, marital status, employment, income, informal social contact,  time variant covariates: self-rated health, functional dependency and number of chronic conditions from previous waves | 3 (baseline 1986; F1 1989; F2 1994)  8 years | Physical health  Self -rated health  Functional dependency  Number of chronic conditions in past 12 months |
| Van Willigen, 2000 (USA) | Community dwelling (≥25 years)  ACL | See Kim & Pai, 2010.  Note: Unable to calculate frequency as responses were all recoded as the midpoint of each response category except the last category which was recoded as 200 hours | age, gender, race, education, marital status, children, employment, income, economic strain, functional impairment, social integration, social support, mastery | 2 (baseline 1986; F1 1989)  3 years | Physical health  Self-rated health  Mental health  Life satisfaction |
| MIDUS (3 papers) | | | | | |
| Choi & Kim, 2011(USA) | Community dwelling (55-84 years at Time 2)  MIDUS I & II  (Baseline age 20-74 years) | Setting: various e.g. hospital, nursing home, or other health related settings, school, other youth-related settings, political organisations or causes, any other organisation, cause or charity  Activity: not reported  Frequency: hours a month  Duration: past 12 months | F1 age, gender, race, ethnicity, education, income, self-rated health, psychological wellbeing, generativity, social integration, number of meetings attended, religiosity | 2 (baseline 1995-6; F1 2004-6)  10 years | Mental health  Psychological wellbeing (Ryff) |

| Fujiwara & Kawachi, 2008 (USA) | Community dwelling (25-74 years)  MIDUS | See Choi & Kim, 2011 | age, gender, race, education, marital status, working status, perceived physical health, depression, extraversion trait, community participation, number of meeting attended, social capital, religiosity | 2 (baseline 1995-6; F1 1998)  2-3 years | Mental health  Major depression (CIDI-SF) |
| --- | --- | --- | --- | --- | --- |
| Son & Wilson, 2012 (USA) | Community dwelling (25-74 years)  MIDUS I & II | See Choi & Kim, 2011 | age, gender, race, marital status, education, income, full time employment, church attendance, physical health | 2 (baseline 1995; F1 2005)  10 years | Mental health  Wellbeing (hedonic, eudemonic and social wellbeing) |
| SHARE (2 papers) | | | | | |
| Siegrist & Wahrendorf, 2009 (Europe^c^) | Community dwelling (≥50 years)  SHARE | Setting: not reported  Activity: formal volunteering and charity work  Frequency: involved, not involved  Duration: past month | age, gender, education, partnership, income, activities of daily living limitations, prior quality of life | 2 (baseline 2004-5; F1 2006-7)  2 years | Mental health  Quality of life (CASP-12) |
| Wahrendorf & Siegrist, 2010 (Europe^d^) | Community dwelling (≥50 years)  SHARE | See Siegrist & Wahrendorf, 2009 | age, gender, education, income, country affiliation, changes in functional health limitations, changes in marital status, changes in retirement status | 2 (baseline 2004-5; F1 2006-7)  2 years | Mental health  Quality of life (CASP-12) |
| WLS (3 papers) | | | | | |
| Konrath et al, 2012 (USA) | Community dwelling high schools graduates in 1957  WLS | Setting: parent and teacher associations (PTAs), youth groups, neighbourhood organisations, community centres, charity or welfare groups  Activity: not reported  Frequency: none, occasionally, when opportunities arose, regularly across some periods, less other times, regularly the whole time  Duration: past 10 years | age, gender, education, marital status, net worth, employment, physical health, functional status, risk factors (e.g. smoking), mental and cognitive health, personality trait, social support, religious attendance volunteering behaviour in past 10 years | 2 (baseline 2004, F1 2008)^b^  4 years | Physical health  Mortality |
| Piliavin, 2005 (USA) | Community dwelling, high school graduates in 1957  WLS | Setting: parent and teacher associations (PTAs), youth groups, neighbourhood organisations, community centres, charity or welfare groups  Activity: not reported  Frequency: constructed trichotomy of longitudinal participation (never volunteered, volunteered in 1 wave, volunteered in both waves)  Duration: not reported | gender, education, children, marital status, IQ, employment, income, social interaction, church attendance, time variant covariates: marital status, employment | 2 (baseline 1975; F1 1992)^b^  17 years | Mental health  Psychological wellbeing (Ryff)  Depression (CES-D) |
| Piliavin & Siegl, 2007 (USA) | Community dwelling, high school graduates in 1957  WLS | See Piliavin, 2005  Constructed dummy variable for diversity of settings (range 1-5) and consistency which indicates the number of waves each participant has volunteered. | gender, IQ, F1 education, F1 marital status, F1 children, head of household, F1 employment, F1 income, F2 health behaviours (body mass index, smoking, exercise), psychological wellbeing, F1, F2 and F3 self orientated organizational involvement , F1 social interactions,  F1 involvement in church | 3 (baseline 1975; F1 1992; F2 2004)^b^  29 years | Physical health  Self-rated health  Mental health  Psychological wellbeing (Ryff) Created indexes combining the 4 scales |

^a^ Comparator = no reported volunteering activity

^b^ The baseline date is the first follow-up that included a question on volunteering, not the original baseline date the original cohort was established.

^c^ Europe: Sweden, Denmark, Netherlands, Germany, Belgium, France, Switzerland, Austria, Italy, Spain, Greece, Poland, Czech Republic, Israel

^d^ Europe: Sweden, Denmark, Netherlands, Germany, Belgium, France, Switzerland, Austria, Italy, Spain, Greece

ACL, Americans’ Changing Lives study; ACS, Alameda County Study; AIM, Aging in Manitoba Study; ALSWH, Australian Longitudinal Study on Women's Health; ELSA, English Longitudinal Study of Ageing; GSOEP, German Socioeconomic Panel; HRS, Health and Retirement Survey; LLSSE, The Later Life Study of Social Exchanges; LSOA, Longitudinal Study of Aging; MIDUS, National Survey of Midlife Development in the United States; MMSA, MacArthur Study of Successful Aging; SHARE, Survey of Health, Ageing and Retirement in Europe; WLS, Wisconsin Longitudinal Study
