# Supplementary material for: Is volunteering a public health intervention? A systematic review and meta-analysis of the health and survival of volunteers
Source: BMC Public Health. 2013 Aug 23;13:773. doi: 10.1186/1471-2458-13-773 (PMC3766013; doi:10.1186/1471-2458-13-773)
Supplement: Additional file 3: Table S3 — The Cochrane Collaboration’s tool for assessing risk of bias (5 trials, 7 papers). [file 1471-2458-13-773-S3.docx]

Table S3 The Cochrane Collaboration’s tool for assessing risk of bias (5 trials, 7 papers)

| **Author, year** | **Random sequence generation** | **Allocation concealment** | **Blinding of personnel^a^** | **Blinding of outcome assessment** | **Incomplete outcome data^b^** | **Selective**  **reporting** | **Free of other bias** | **Overall risk of bias^c^** |
| --- | --- | --- | --- | --- | --- | --- | --- | --- |
| Cohen, 2009 | Unclear | Unclear | Unclear | Unclear | Unclear | Low | High - small sample size | High |
| Experience Corps^d^ (refers to Carlson et al, 2008, Fried et al, 2004 and Tan et al, 2006) | Unclear | Unclear | Low | Low | Low | Low | High - small sample size | Moderate |
| George & Singer, 2011 | Low | Unclear | Unclear | Unclear | Low | Low | High - small sample size | Low |
| Rook & Sorkin, 2003^e^ | Unclear | Unclear | Low | Low | High | Low | High - small sample size | High |
| Yuen et al, 2008 | Unclear | Unclear | High | High | High | Low | High - small sample size | High |

^a^ Impossible to blind participants to group allocation. Current application of the Cochrane criteria assumes that lack of participant blinding is problematic, but this is a flexible judgment according to the guidance notes.

^b^ A loss to follow-up (excluding death) of >20% in either group, or in the cohort as a whole was defined as evidence of unacceptably high attrition.

^c^ The two domains ‘random sequence generation’ and ‘incomplete outcome data’ were prioritised as non-representative samples and high attrition rates are considered major threats to the external validity of community based studies. The overall risk of bias score was generated for each study using the scores for these two domains:

High risk of bias either domain not addressed (both at high risk or unclear)

Moderate risk of bias one prioritised domain addressed (low risk) while the other not addressed (high risk or unclear)

Low risk of bias both prioritised domains addressed (low risk)

^d^ Given the EC papers appeared to be reporting different outcomes but from the same cohort of participants, the quality of the EC papers was scored as one cohort by deriving details from each paper.

^e^ The community sample arm of the trial which did not include randomisation was excluded from our data synthesis.
