# Supplementary material for: Is volunteering a public health intervention? A systematic review and meta-analysis of the health and survival of volunteers
Source: BMC Public Health. 2013 Aug 23;13:773. doi: 10.1186/1471-2458-13-773 (PMC3766013; doi:10.1186/1471-2458-13-773)
Supplement: Additional file 4: Table S4 — NOS scores of non-RCTs and longitudinal cohort studies (4 non-RCTs, 17 unique cohorts, 33 papers). [file 1471-2458-13-773-S4.docx]

Table S4 NOS scores of non-RCTs and longitudinal cohort studies (4 non-RCTs, 17 unique cohorts, 33 papers)

| **Authors, year** | **Selection** | | | | | **Comparability** | | **Outcome** | | | | | **Risk score** |
| --- | --- | --- | --- | --- | --- | --- | --- | --- | --- | --- | --- | --- | --- |
|  | 1a) Intervention group truly representative | 1b) Intervention group somewhat representative | 2a) Control group from same community | 3a) Secure record used | 3b) Structured interview used | 1a) Controlled for important factor | 1b) Controlled for additional factor | 1a) Blind assessment | 1b) Record linkage | 2a) Sufficient follow-up period | 3a) Complete follow-up | 3b) Loss to follow-up unlikely to affect bias | High risk (0-3)  Moderate risk (4-5)  Low risk (6-8) |
| **Non-RCTs (4 controlled trials)** | | | | | | | | | | | | | |
| Belgrave, 2011 | - | - | √ | √ | - | - | - | - | - | √ | √ | - | Moderate (4) |
| Dabelko-Schoeny et al, 2010 | - | - | √ | √ | - | √ | - | - | - | - | √ | - | Moderate (4) |
| Carlson et al, 2009^a^ | - | - | √ | √ | - | √ | √ | - | √ | √ | - | √ | Low (7) |
| Tan et al, 2009^a^ | - | - | - | √ | - | √ | √ | - | - | √ | √ | - | Moderate (5) |
| **Cohorts (17 unique cohorts, 29 papers)** | | | | | | | | | | | | | |
| Ayalon, 2008 | √ | - | √ | - | √ | √ | √ | - | √ | √ | - | √ | Low (8) |
| Bowman et al, 2010 | - | - | √ | - | - | √ | √ | - | - | √ | √ | - | Moderate (5) |
| Choi & Bohman, 2007 | - | √ | √ | - | √ | √ | √ | - | - | - | - | √ | Low (6) |
| Harris & Thoresen, 2005 | √ | - | √ | - | - | √ | √ | - | √ | √ | - | √ | Low (7) |
| Jung et al, 2010 | - | - | √ | - | √ | √ | √ | - | - | √ | √ | - | Low (6) |
| Meier & Stutzer, 2008 | - | √ | √ | - | - | √ | √ | - | - | √ | - | - | Moderate (5) |
| Menec, 2003 | - | - | √ | - | √ | √ | √ | - | - | √ | √ | - | Low (6) |
| Moen et al, 1992 | - | - | √ | - | √ | √ | √ | - | - | √ | - | √ | Low (6) |
| Nazroo & Matthews, 2012 | - | √ | √ | - | - | √ | √ | - | - | √ | √ | - | Low (6) |
| Okun et al, 2010 | - | √ | √ | - | √ | √ | √ | - | √ | √ | √ | - | Low (8) |
| Oman et al, 1999 | - | √ | √ | - | - | √ | √ | - | √ | √ | √ | - | Low (7) |
| Pillemer et al, 2010 | - | √ | √ | - | - | √ | √ | - | - | √ | √ | - | Low (6) |
| Shimanuki et al, 2007 | - | - | √ | √ | - | √ | √ | - | - | √ | √ | - | Low (6) |
| ACL (8 papers) | | | | | | | | | | | | | |
| Kim & Pai, 2010 | - | √ | √ | - | - | √ | √ | - | - | √ | - | √ | Low (6) |
| Li & Ferraro, 2005 | - | √ | √ | - | √ | √ | √ | - | - | √ | √ | - | Low (7) |
| Li & Ferraro, 2006 | - | √ | √ | - | √ | √ | √ | - | - | √ | - | √ | Low (7) |
| Morrow-Howell et al, 2003 | - | √ | √ | - | √ | √ | √ | - | - | √ | - | √ | Low (7) |
| Musick & Wilson, 2003 | - | √ | √ | - | √ | √ | √ | - | - | √ | - | - | Low (6) |
| Musick et al, 1999 | - | √ | √ | - | - | √ | √ | - | √ | √ | - | √ | Low (7) |
| Tang, 2009 | - | √ | √ | - | √ | √ | √ | - | - | √ | - | - | Low (6) |
| Van Willigen, 2000 | - | √ | √ | - | - | √ | √ | - | - | √ | - | - | Moderate (5) |
| MIDUS (3 papers) | | | | | | | | | | | | | |
| Choi & Kim, 2011 | - | - | √ | - | - | √ | √ | - | - | √ | √ | - | Moderate (5) |
| Fujiwara & Kawachi, 2008 | - | √ | √ | - | √ | √ | √ | - | - | √ | √ | - | Low (7) |
| Son & Wilson, 2012 | - | √ | √ | - | - | √ | √ | - | - | √ | - | √ | Low (6) |
| SHARE (2 papers) | | | | | | | | | | | | | |
| Siegrist & Wahrendorf, 2009 | - | √ | √ | - | √ | √ | √ | - | - | √ | √ | - | Low (7) |
| Wahrendorf & Siegrist, 2010 | - | √ | √ | - | - | √ | √ | - | - | √ | √ | - | Low (6) |
| WLS (3 papers) | | | | | | | | | | | | | |
| Konrath et al, 2012 | - | √ | √ | - | - | √ | √ | - | √ | - | √ | - | Low (6) |
| Piliavin, 2005 | - | √ | √ | - | - | √ | √ | - | - | √ | √ | - | Low (6) |
| Piliavin & Siegl, 2007 | - | √ | √ | - | - | √ | √ | - | - | √ | √ | - | Low (6) |

Shaded columns highlight mutually exclusive components of each assessment. A tick represents a point scored.

^a^ These two papers report very different experiments using Experience Corp participants and are therefore presented separately.

ACL, Americans’ Changing Lives study; EC, Experience Corps Program; MIDUS, National Survey of Midlife Development in the United States; SHARE, Survey of Health, Ageing and Retirement in Europe; WLS, Wisconsin Longitudinal Study
