# Supplementary material for: Is volunteering a public health intervention? A systematic review and meta-analysis of the health and survival of volunteers
Source: BMC Public Health. 2013 Aug 23;13:773. doi: 10.1186/1471-2458-13-773 (PMC3766013; doi:10.1186/1471-2458-13-773)
Supplement: Additional file 5: Table S5 — Vote counting for experimental study designs (9 trials, 11 papers). [file 1471-2458-13-773-S5.docx]

**Table S5 Vote counting^a^ for experimental study designs (9 trials, 11 papers)**

| Authors, year | N participants randomised  (n allocated to intervention, control) | N participants at final follow-up (n allocated to intervention, control) | Baseline mean age or range (years) | Baseline gender  (% female) | Physical activity | Self-rated health | Depression | Life satisfaction | Self-esteem | Cognitive function | Other outcomes |
| --- | --- | --- | --- | --- | --- | --- | --- | --- | --- | --- | --- |
| RCT (5 trials, 7 papers) | | | | | | | | | | | |
| Cohen, 2009 | 70 (35, 35) | 70 (35, 35) | Overall NR  41.6 (I)  41.8 (C) | Overall NR  60.0% (I)  54.5% (C) | - | - | - | - | - | - | √ Empowerment (composite of critical awareness, self-efficacy and interpersonal and political skills) |
| George & Singer, 2011 | 16 (8, 8) | 15 (8, 7) | Overall NR  85.7 (I)  81.4 (C) | 86.7% overall  87.5% (I)  85.7% (C) | - | - | = | - | - | = | √ Stress  = Sense of purpose  = Sense of usefulness |
| Rook & Sorkin, 2003 | 121 (52, 69) | 72 (20, 52) | Overall NR  69.6 (I)  68.9 (C) | Overall NR  67.3% (I)  69.6% (C) | - | - | = F1  = F2 | - | = F1  = F2 | - | = Loneliness (F1, F2) |
| Yuen et al, 2008 | 39 (19, 20) | 26 (13, 13) | 83.4 overall  83.0 (I)  83.9 (C) | 71.4% overall 80.0 % (I)  61.5% (C) | - | √ F1  =F2 | = F1  = F2 | = F1  = F2 | - | - | √ Wellbeing^b^ |
| EC (3 papers) | | | | | | | | | | | |
| Carlson et al, 2008 | 149 (70, 58)  21 withdrew post randomisation | 110 ( 62,48) | 69 .0 overall  70.1 (I)  68.4 (C) | 90.0% overall  83.0% (I)  93.0% (C) | - | - | - | - | - | √ (3/6 outcomes)^c^ | - |
| Fried et al, 2004 | 148 (70, 58)  20 withdrew post randomisation | 125 (69, 56) | 69.0 overall  (I and C NR) | 91.7% overall  88.6% (I)  94.8% (C) | √ (1/7 outcomes) | - | - | - | - | √ (1/5 outcomes)^d^ | √ Strength  = Falls in last year  = Cane use  √ Walking speed |
| Tan et al, 2006 | 148 (70, 58)  20 withdrew post randomisation | 113 (59, 54) | 69.0 overall  (I and C NR) | 94%  92% (I)  96% (C) | =^e^ | - | - | - | - | - | - |
| Non-RCTs (4 trials) | | | | | | | | | | | |
| Belgrave, 2011 | 27 | 26 (14, 12) | NR - retirement age | NR | - | - | - | - | = | - | - |
| Dabelko-Schoeny et al, 2010 | 54 (i and c NR) | 43 (22, 21) | Overall NR  77.0 (I)  76.0 (C) | Overall NR  82% (I)  76% (C) | - | = | - | - | = | - | = Purpose in life |
| Carlson et al, 2009^f^ | 18 (9, 9) | 17 (8,9) | Overall NR  68.0 (I)  67.8 (C) | 100% overall | - | - | - | - | - | √ | - |
| Tan et al, 2009^f^ | 221 (71, 150) | 221 (71, 150) | Overall NR  71.2 (I)  73.0 (C) | 100% overall | √ | - | - | - | - | - | - |

^a^For each outcome, studies were categorised as either a statistically significant benefit (with p≤0.05) in favour of volunteering (√), no difference between groups (=), a statistically significant negative effect (with p≤0.05) of volunteering (X), or a dash (-) if the outcome was not used.

^b^ Wellbeing was a composite score using self-rated health, depression and life satisfaction data in a multivariate nonparametric global statistical test. However, there were sufficient data for each component to calculate the impact of volunteering.

^c^Sub group analysis stratified by baseline impairment (or not) suggests effects stronger in impaired sub group.

^d^ Outcomes were self-reported cognitive activities outside the study which were different to those outcomes measured within the study by Carlson et al, 2008.

^e^ Sub group analysis stratified by baseline activity (high or low) suggests significant effects in low activity sub group in 3/7 outcomes.

^f^ These two papers report very different experiments using Experience Corp data and are therefore presented separately.

C, control group; F, follow- up; I, intervention group; NR, not reported
