# Supplementary material for: Is volunteering a public health intervention? A systematic review and meta-analysis of the health and survival of volunteers
Source: BMC Public Health. 2013 Aug 23;13:773. doi: 10.1186/1471-2458-13-773 (PMC3766013; doi:10.1186/1471-2458-13-773)
Supplement: Additional file 6: Table S6 — Vote counting for longitudinal study designs (17 unique cohorts, 29 papers). [file 1471-2458-13-773-S6.docx]

**Table S6 Vote counting^a^ for longitudinal study designs (17 unique cohorts, 29 papers)**

| **Authors, year** | **N at original cohort baseline** | **N in data analyses**  **(n or % allocated to intervention, control)** | **Mean age or range (years)** | **Gender**  **(% female)** | **Mortality** | **Functional ability** | **Self-rated health** | **Depression** | **Life satisfaction** | **Wellbeing** | **Quality of life** | **Other outcome** |
| --- | --- | --- | --- | --- | --- | --- | --- | --- | --- | --- | --- | --- |
| Unique cohorts (n=13) | | | | | | | | | | | | |
| Ayalon, 2008 | 5055 | 5055  (10.7%, 89.2%) | Overall range: 60-80+ years | Overall NR  51.9% (I)  56.5% (C) | √ vol status  √ hours  √ years vol | - | - | - | - | - | - | - |
| Bowman et al, 2010 | 1542 | 416  (I and C NR) | Overall range: 17-19 years at baseline | 43% | - | - | - | - | √ | √ | - | - |
| Choi & Bohman, 2007 | 11111 | 8030  (33.5% female 31.7% male, 66.5% female 68.3% male) | Overall range: 65-85+ years | 60.7%  (I and C NR) | - | - | - | vol status  √ women  = men | - | - | - | - |
| Harris & Thoresen, 2005 | 7527 | 7496  (15.4%, 84.6%) | 76.8  (I and C NR) | 62.1%  (I and C NR) | √ frequent vol  = rarely/ sometimes vol | - | - | - | - | - | - | - |
| Jung et al, 2010 | 1189 | 1072  (28.0%, 72.0%) | Overall range: 70-79 years | NR | - | - | - | - | - | - | - | Frailty  = vol status  = hours |
| Meier & Stutzer, 2008 | 22016 | 22016  (23.0%, 77.0%) | NR | NR | - | - | - | - | √ vol status  √ intensity:  weekly only | - | - | - |
| Menec, 2003 | 3218 | 1439-2291  (22.9%, 77.1%) | 75.7 overall  (I and C NR) | 61.1% overall  (I and C NR) | = | √ | - | - | = | - | - | Happiness  = vol status |
| Moen et al, 1992 | 427 | 270-284  (75.6%, 24.4%) | In 1956: 35.3  (I and C NR) | 100% overall | - | = sustained vol  √ intermittent vol  = age of starting or stopping vol | = | - | - | - | - | - |
| Nazroo & Matthews, 2012 | NR | 3632  (I and C NR) | Overall range:  60-80+ female  65-80+ male | NR | - | - | - | √ vol status  √ reciprocal activity  = non-reciprocal activity  √ sustained vol  = intermittent vol | √ vol status  √ reciprocal activity  = non-reciprocal activity  √ sustained vol  = intermittent vol | - | √ vol status  √ reciprocal activity  = non-reciprocal activity  = sustained vol  = intermittent vol | - |
| **Authors, year** | **N at original cohort baseline** | **N in data analyses**  **(n or % allocated to intervention, control)** | **Mean age or range (years)** | **Gender**  **(% female)** | **Mortality** | **Functional ability** | **Self-rated health** | **Depression** | **Life satisfaction** | **Wellbeing** | **Quality of life** | **Other outcomes** |
| Okun et al, 2010 | 916 | 868 (31%, 69%) | 74.1 overall  (I and C NR) | 62% overall  (I and C NR) | = vol status  = vol frequency | - | - | - | - | - | - | - |
| Oman et al, 1999 | 2025 | 1973-2021  (630, 1391) | Overall range: 55-85+ years | 58%  (I and C NR) | = vol status  √ ≥2 orgs | - | - | - | - | - | - | - |
| Pillemer et al, 2010 | 6928 | 2630  (115 environmental volunteers 1186 other volunteers 1289 non volunteers) | 44.7 overall  (I and C NR) | 56.9% overall  (I and C NR) | - | - | √ environ-mental vol  = other vol | = | - | - | - | Physical activity  √ vol status |
| Shimanuki et al, 2007 | 1503 | 1276  (69, 1207) | overall NR  73.0 (I)  75.5 (C) | overall NR  26.1% (I)  63.1% (C) | - | = | - | - | - | - | - | Self-efficacy for ADL  √ vol status |
| ACL (8 papers) | | | | | | | | | | | | |
| Kim & Pai, 2010 | 3617 | 3,617  (39.9%, 60.1%) | 53.6  (I and C NR) | 62.4%  (I and C NR) | - | - | - | vol status and hours:  = <65 yrs  √ 65+ yrs | - | - | - | - |
| Li & Ferraro, 2005 | 1669 | 815  (I and C NR) | 67.6  (I and C NR) | 71%  (I and C NR) | - | - | - | √ | - | - | - | - |
| Li & Ferraro, 2006 | 2544 | 1446: middle age: 624 older age: 822 (I and C NR) | Overall: middle age: 49.0 older aged: 67.6  (I and C NR) | Overall: middle age: 63% older aged: 71%  (I and C NR) | - | = 40-59 yrs  √ 60+ yrs | - | = 40-59 yrs  √ 60+ yrs | **-** | **-** | **-** | **-** |
| Morrow-Howell et al, 2003 | 1669 | 1669  (34.5%, 65.5%) | 70.1 overall  (I and C NR) | 67.0% overall  (I and C NR) | - | √ vol status  √ hours  = nos orgs  √ religious orgs  = other orgs | √ vol status  √ hours  = nos orgs  = type org | √ vol status  √ hours  = nos orgs  = type org | - | - | - | - |
| Musick & Wilson, 2003 | 3617 | 3617  (25% church, 23% secular only, 52%) | 44.4 overall  46.0 church + other vol, 41.2 secular vol only  45.0 (C) | 52% overall  57% church + other vol,  54% secular vol only,  50%(C) | - | - | - | Irrespective of org type:  = <65 yrs  √ 65+ yrs  sustained vol  = <65 yrs  √ 65+ yrs | - | - | - | - |
| **Authors, year** | **N at original cohort baseline** | **N in data analyses**  **(n or % allocated to intervention, control)** | **Mean age or range (years)** | **Gender**  **(% female)** | **Mortality** | **Functional ability** | **Self-rated health** | **Depression** | **Life satisfaction** | **Wellbeing** | **Quality of life** | **Other outcomes** |
| Musick & Wilson, 2003 | 3617 | 3617  (25% church, 23% secular only, 52%) | 44.4 overall  46.0 church + other vol, 41.2 secular vol only  45.0 (C) | 52% overall  57% church + other vol,  54% secular vol only,  50%(C) | - | - | - | Irrespective of org type:  = <65 yrs  √ 65+ yrs  sustained vol  = <65 yrs  √ 65+ yrs | - | - | - | - |
| Musick et al, 1999 | 1211 | 1211  (34.0%, 66.0%) | 73.0 overall  (I and C NR) | 60% overall  (I and C NR) | √ 1 org  = >1org  = hours | - | - | - | - | - | - | - |
| Tang, 2009 | 1669 | 1669  (34.5%, 65.5%) | 70.1  (I and C NR) | 67.1% overall  (I and C NR) | - | √ vol status  √ hours | √ vol status  √ hours | - | - | - | - | Number of chronic conditions  = vol status  = hours |
| Van Willigen, 2000 | 3617 | 2867  Volunteers:  younger 1052 (37%), older 278 (10%)  Non-volunteers:  younger 1110 (38%), older 427 (15%) | Overall NR  Volunteers:  25-59 yrs: 38.7  ≥60 yrs: 68.7  Non-volunteers:  25-59 yrs: 39.2  ≥60 yrs: 70.2 | Overall NR  Volunteers:  25-59 yrs: 54.0%  ≥60 yrs: 59.0%  Non-volunteers  25-59 yrs: 48.0%  ≥60 yrs: 61.0% | - | - | √ vol status  √ 1 org  = >1 orgs 25-59 yrs  √ >1 orgs ≥60 yrs  √ hours | - | √ vol status  √ 1 org  √ >1 orgs  √ hours | - | - | - |
| MIDUS (3 papers) | | | | | | | | | | | | |
| Choi & Kim, 2011 | 1672 | 878-917  (35.6%, 60.9%) (3.5% missing) | Overall range: 55-84 years | 54.0%  (I and C NR) | - | - | - | - | - | √ 1-10 hrs/ month of vol  = 11+ hrs/ month vol | - | - |
| Fujiwara & Kawachi, 2008 | 724 | Weighted: 610-628  (38.4%, 61.6%) | Overall range: 25-74 years | 56.0% (weighted)  (I and C NR) | - | - | - | = | - | - | - | - |
| Son & Wilson, 2012 | 3487 | 3257  (39%, 61%) | 42.8  (I and C NR) | 55%  (I and C NR) | - | - | - | - | - | Social and eudemonic:  √ vol status  = hours  Hedonic:  = vol status  = hours | - |  |
| **Authors, year** | **N at original cohort baseline** | **N in data analyses**  **(n or % allocated to intervention, control)** | **Mean age or range (years)** | **Gender**  **(% female)** | **Mortality** | **Functional ability** | **Self-rated health** | **Depression** | **Life satisfaction** | **Wellbeing** | **Quality of life** | **Other outcomes** |
| SHARE (2 papers) | | | | | | | | | | | | |
| Siegrist & Wahrendorf, 2009 | 14517 | 8896  (12.4%, 87.61%) | Overall range: 50-75+ years | 46.3%  (I and C NR) | - | - | - | - | - | - | √ reciprocal activity  = non-reciprocal activity | - |
| Wahrendorf & Siegrist, 2010 | 10309 | 10309  (836 continuous volunteer; 617 giving up  716 starting  8140 non-volunteer) | Overall range: 50-75+ years | 53.1% overall  (I and C NR) | - | - | - | - | - | - | √ vol status | - |
| WLS (3 papers) | | | | | | | | | | | | |
| Konrath et al, 2012 | 5512^b^ | 5512  (I and C NR) | 69.2  (I and C NR) | 51.6%  (I and C NR) | = | - | - | - | - | - | - | - |
| Piliavin, 2005 | 10317^c^ | 6900 in 1992  (I and C NR) | Early 50s  (I and C NR) | 53.6% overall  (I and C NR) | - | - | - | √ sustained vol  = intermittent vol | - | √ sustained vol  √ intermittent vol  √ ≥1 orgs | - | - |
| Piliavin & Siegl, 2007 | 10317^c^ | 4000  (I and C NR) | NR | 53.6% overall  (I and C NR) | - | - | √ vol status  √ sustained vol  √ ≥1 orgs | - | - | √ vol status  √ sustained vol  √ ≥1 orgs | - | - |

^a^ For each outcome, studies were categorised as either a statistically significant benefit (with p≤0.05) in favour of volunteering (√), no difference between groups (=), a statistically significant negative effect (with p≤0.05) of volunteering (X), or a dash (-) if the outcome was not used.

^b^ Total number of participants in 2004 not reported, just the number of respondents (n=5512)

^c^ Cohort formed in 1957 recruiting 10317 participants. Volunteering status was not measured until 1975 which forms the baseline for this synthesis, although the number of participants contributing data at this point is not reported.

ADL, activities of daily living; C, control group; I intervention group; NR, not reported; org, organisation; vol volunteering
